# *K*-Profiles: A Nonlinear Clustering Method for Pattern Detection in High Dimensional Data

**DOI:** 10.1155/2015/918954

**Published:** 2015-08-03

**Authors:** Kai Wang, Qing Zhao, Jianwei Lu, Tianwei Yu

**Affiliations:** ^1^Department of Mathematics and Computer Science, Emory University, Atlanta, GA 30322, USA; ^2^School of Software Engineering, Tongji University, Shanghai 200092, China; ^3^The Advanced Institute of Translational Medicine and Department of Gastroenterology, . Shanghai Tenth People's Hospital, Tongji University, Shanghai 200092, China; ^4^Department of Biostatistics and Bioinformatics, Emory University, Atlanta, GA 30322, USA

## Abstract

With modern technologies such as microarray, deep sequencing, and liquid chromatography-mass spectrometry (LC-MS), it is possible to measure the expression levels of thousands of genes/proteins simultaneously to unravel important biological processes. A very first step towards elucidating hidden patterns and understanding the massive data is the application of clustering techniques. Nonlinear relations, which were mostly unutilized in contrast to linear correlations, are prevalent in high-throughput data. In many cases, nonlinear relations can model the biological relationship more precisely and reflect critical patterns in the biological systems. Using the general dependency measure, Distance Based on Conditional Ordered List (DCOL) that we introduced before, we designed the nonlinear *K*-profiles clustering method, which can be seen as the nonlinear counterpart of the *K*-means clustering algorithm. The method has a built-in statistical testing procedure that ensures genes not belonging to any cluster do not impact the estimation of cluster profiles. Results from extensive simulation studies showed that *K*-profiles clustering not only outperformed traditional linear *K*-means algorithm, but also presented significantly better performance over our previous General Dependency Hierarchical Clustering (GDHC) algorithm. We further analyzed a gene expression dataset, on which *K*-profile clustering generated biologically meaningful results.

## 1. Introduction

In recent years, large amounts of high dimensional data have been generated from high-throughput expression techniques, such as gene expression data using microarray or deep sequencing [1], and metabolomics and proteomics data using liquid chromatography-mass spectrometry (LC-MS) [2]. Mining the hidden patterns inside these data leads to an enhanced understanding of functional genomics, gene regulatory networks, and so forth [3, 4]. However, the complexity of biological networks and the huge number of genes pose great challenges to analyze the big mass of data [5, 6]. Clustering techniques has usually been applied as a first step in the data mining process to analyze hidden structures and reveal interesting patterns in the data [7].

Clustering algorithms have been studied extensively in the last three decades, with many traditional clustering techniques successfully applied or adapted to gene expression data, which led to the discovery of biologically relevant groups of genes or samples [6]. Traditional clustering algorithms usually process data on the full feature space while emerging attention has been paid to subspace clustering. Traditional clustering algorithms, such as *K*-means and expectation maximization (EM) based algorithms, mostly use linear associations or geometric proximity to measure the similarity/distance between data points [8].

When applying traditional clustering algorithms to the domain of bioinformatics, additional challenges are faced due to prevalent existence of nonlinear correlations in the high dimensional space [9]. However, nonlinear correlations are largely untouched in contrast to the relative mature literature of clustering using linear correlations [5, 10–12]. There are several factors making nonlinear clustering difficult. First, a pair of nonlinearly associated data points may not be close to each other in high-dimensional space. Second, it is difficult to effectively define a cluster profile (i.e., the “center” of a cluster) to summarize a cluster given the existence of nonlinear associations. Third, compared to measures that detect linear correlations, nonlinear association measures lose statistical power more quickly with the increase of random additive noise. Fourth, given the high dimensions, computationally expensive methods, for example, principal curves [13, 14], are hard to be adopted even though they can model nonlinear relationships.

In this paper, we try to address these problems by developing a clustering method that can group data points with both linear and nonlinear associations. We name this method “*K*-profiles clustering.” Our method is based on the previously described nonlinear measure: the Distance Based on Conditional Ordered List (DCOL) [15, 16]. The key concept is to use data point orders in the sample space as the cluster profile. We have previously described a hierarchical clustering scheme named General Dependency Hierarchical Clustering (GDHC). However the computation of GDHC is very intensive. The new *K*-profiles clustering method is much more efficient, representing a ~20-fold reduction in computing time. Conceptually, it is the nonlinear counterpart of the popular *K*-means clustering method, while the existing GDHC is the nonlinear counterpart of the traditional hierarchical clustering method. Another key advantage of the *K*-profiles clustering method is that, by building statistical inference into the iterations, noise genes that do not belong to any cluster will not interfere with the cluster profile estimation, and they are naturally left out of the final results.

## 2. Methods

### 2.1. Distance Based on Conditional Ordered List (DCOL)

We first consider the definition of Distance Based on Conditional Ordered List (DCOL) in two-dimensional space. Given two random variables *X* and *Y* and the corresponding data points {(*x*_*i*_, *y*_*i*_)}_*i*=1,…,*n*_, after sorting the points on *x*-axis to obtain

(1) $$\begin{array}{c} \left\{\left(x_{i}^{*},y_{i}^{*}\right):x_{1}^{*}\leq x_{2}^{*}\leq \cdots \leq x_{n}^{*}\right\} \end{array}$$

the DCOL is defined as

(2) $$\begin{array}{c} d_{col}\left(Y\;|\;X\right)=\frac{1}{\left(n-1\right)}\overset{n}{\underset{i=2}{\sum }}\left|y_{i}-y_{i-1}\right|. \end{array}$$

Intuitively, when *Y* is less spread in the order sorted on *X*, *d*_col_(*Y*∣*X*) is small. We can use *d*_col_(*Y*∣*X*) to measure the spread of conditional distribution *Y*∣*X* in a nonparametric manner [16].

The statistical inference on *d*_col_(*Y*∣*X*) can be conducted using a permutation test. Under the null hypothesis that *X* and *Y* are independent of each other, the ordering of the data points based on *X* is simply a random reordering of *Y*. Thus we can randomly permute {(*y*_*i*_)}_*i*=1,…,*n*_ *B* times and calculate the sum of distances between adjacent *Y* values in each permutation. Then we can find the mean and standard deviation from the *B* values sampled from the null distribution. The actual *d*_col_(*Y*∣*X*) can then be compared to the estimated null distribution to obtain the *p* value. Notice this process does not depend on *X*. The permutation can be done once for *Y* and the resulting null distribution parameters apply to any *X*, which greatly saves computing time.

### 2.2. Defining a Cluster Profile and Generalizing DCOL to Higher Dimensions

Let **U** be a *p*-dimensional random vector (*X*_1_, *X*_2_,…, *X*_*p*_), where each *X*_*i*_ is a random variable; then an instance of random vector **U** can be seen as a point in the *p*-dimensional space. Assuming instances of random vector **U** are sorted in the *p*-dimensional space, then *d*_col_(*Y*∣**U**) can be computed according to (2) for any random variable *Y*. Therefore, the key problem is to define the order of a series of *p*-dimensional points.

When *X* is one-dimensional, we can easily prove that a list of numbers (*x*_1_, *x*_2_,…, *x*_*n*_) is sorted if and only if ∑_*i*=2_^*n*^|*x*_*i*_ − *x*_*i*+1_| is minimized. We generalize this to *p*-dimensional space and define instances (**u**_1_, **u**_2_,…, **u**_*n*_) as sorted if and only if the sum of distances between the adjacent *p*-dimensional points is minimized. Sorting the points is equivalent to finding the shortest Hamiltonian path through the *n* points in *p* dimensions, the solution of which is linked to the Traveling Salesman Problem (TSP) [17]. Many methods exist for solving the TSP [17].

If we consider the *p* random variables as *p* genes, we have effectively defined a profile for the cluster made of these *p* genes. Using this profile, we can compute the *d*_col_(*Y*∣**U**) for any gene *Y* and determine if the gene is close to this cluster, which serves as the foundation of the *K*-profile algorithm.

### 2.3. The **K**-Profiles Algorithm

In this section, we outline the DCOL-based nonlinear *K*-profiles clustering algorithm. First, we define the gene expression data matrix *G*_*p*×*n*_, where *n* samples are measured for *p* genes and each cell *g*_*ij*_ is the measured expression level of gene *i* on sample *j*. Each row represents the expression pattern of a gene while each column represents the expression profile of a specified sample.

The *K*-profiles clustering process is analogous to the traditional *K*-means algorithm overall. However there are two key differences: (1) Different from the *K*-means clustering algorithm, we use the data point ordering (Hamiltonian path) as the cluster profile rather than the mean vector of all data points belonging to this cluster; (2) during the iterations, the association of each point to its closest cluster is judged for statistical significance. Points that are not significantly associated with any cluster cannot contribute to the estimation of the cluster's profile.

Due to the random initialization of clusters, we use a loose *p* value cutoff at the beginning and decrease it iteration by iteration as the updated cluster profiles become more stable and reflect the authentic clusters more reliably as the clustering process progresses.

(a) To start, we compute the null distribution of DCOL distances for each gene (row) and obtain two parameters, mean *μ*_*i*_ and standard deviation *σ*_*i*_, for each gene simultaneously by permuting columns of the matrix 500 times. The gene-specific null distribution parameters are used to compute the *p* values of the DCOL whenever assigning a gene to the closest cluster.
(b) Initialize *K* clusters by generating *K* random orders as cluster profiles; set *p* value cutoff to upper bound.
(c) For each row vector, compute its DCOL distance to each cluster according to corresponding cluster profile *d*_col_(*X*_*i*_∣**U**_*k*_), where *X*_*i*_ is the *i*th gene and **U**_*k*_ is the *k*th cluster. Assign it to the closest cluster if the DCOL is statistically significant in terms of *p* value. In this step, we are implicitly computing *K* *p* values for each gene and taking the minimum. Thus we need to adjust the *p* value cutoff to address the multiple testing issue. We assume each cluster profile is independent of the others. Then it follows that, for each gene, the *K* *p* values are independent. Under the null hypothesis that the gene is not associated with any of the clusters, all the *p* values are* i.i.d*. samples from the standard uniform distribution. Thus the nominal *p* value cutoff *π* is transformed to *π*′ = 1 − (1 − *π*)^1/*K*^.
(d) When all gene vectors have been assigned, recalculate the profile of each cluster using a TSP solver.
(e) Repeat steps (c) and (d) until the cluster profiles no longer change or the maximum iteration is reached. We start with a loose *p* value cutoff. In each iteration we reduce the *p* value cutoff by a small amount, until the target *p* value cutoff is reached.

The above procedure is conditioned on a given *K*, the number of clusters. We used gap statistics for determination of *K*. Other options such as prediction strength or finding the elbow of the variance-cluster number plot are also available. Here we replace the sum of variances by the sum of negative log*p* values.

### 2.4. Simulation Study

**Figure 1:**
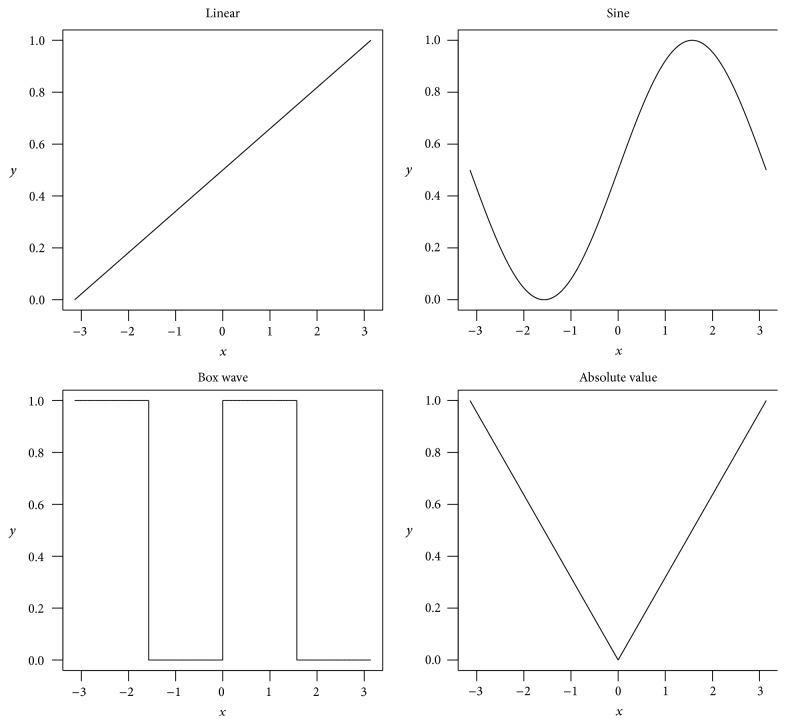
Illustration of the four functions used in simulations.

We generated simulation datasets with 100 samples (columns) and *M* gene clusters, each containing 100 genes (rows). Another 100 pure noise genes were added to the data. *M* was set to 10 or 20 in separate simulation scenarios. Within each cluster, we set the genes (rows) to be either linearly or nonlinearly correlated using different link functions, including (1) linear, (2) sine curve, (3) box wave, and (4) absolute value (Figure 1).

Clusters were generated separately using three different mechanisms, namely, (1) the hidden factor data generation approach, (2) 1-dependent approach, and (3) 2-dependent approach.

In the hidden factor approach, for each cluster, we first generated the expression levels of a single controlling factor *z* by sampling the standard normal distribution. Then for each gene, a function was randomly drawn from the four functions mentioned above (Figure 1). The gene was generated as the function of the hidden controlling factor plus certain level of noise from the normal distribution: *x* ^(new)^ = *f*(*z*) + *ε*.

In the 1-dependent approach, the expressions of genes in a cluster were generated sequentially. The first gene was generated by sampling the standard normal distribution. From the second gene on, we first randomly chose one gene that was already generated and randomly chose one function from the four available functions (Figure 1). We then generated the new gene as the function of the previously generated gene: *x* ^(new)^ = *f*(*x* ^(selected)^). After the expression of all genes in a cluster was generated, certain level of noise was generated from the normal distribution and added to the gene expression profiles.

The 2-dependent approach is similar to the 1-dependent approach. The difference is that, for each new gene, two previously generated genes were randomly selected, and two functions were randomly chosen. The new gene was generated as the summation: *x* ^(new)^ = *β*_1_ *f*(*x* ^(selected_1)^) + *β*_2_ *g*(*x* ^(selected_2)^). The *β*'s were sampled from the uniform distribution between −1 and 1. Again certain level of noise was generated from the normal distribution and added to the gene expression profiles.

## 3. Results and Discussions

### 3.1. Simulation Results

In the simulation experiments, we compared the *K*-profiles algorithm with General Dependency Hierarchical Clustering (GDHC) and the traditional *K*-means clustering algorithm. The GDHC was paired with the dynamic tree cutting method to cut the trees into clusters [18]. We used the efficient TSP R library to compute the cluster profiles [19]. We adopted the external evaluation metric Adjusted Rand Index (ARI) [20] to compare the clustering results with the true cluster memberships to judge the performance of the methods.

**Figure 2:**
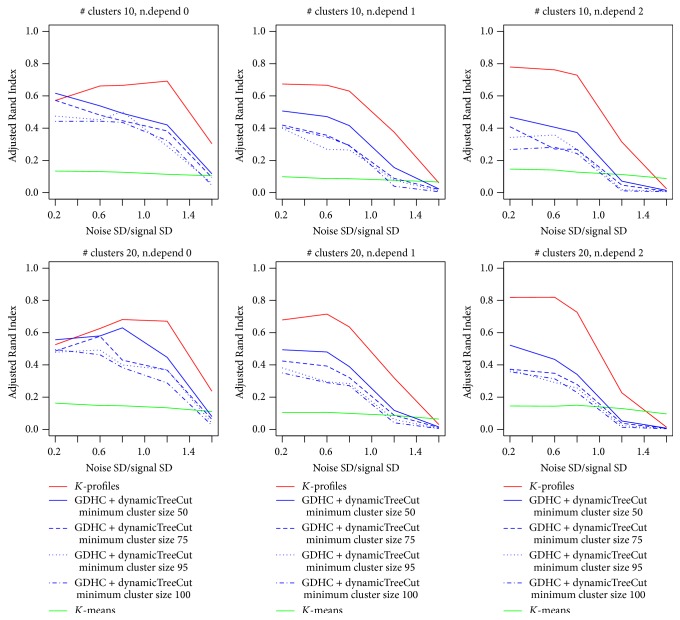
Simulation results with nonlinear data.

In Figure 2, the average ARI values were plotted against the noise level. Higher ARI values indicate better clustering performance. The figure contains three columns and two rows with each column representing a data generation mechanism and each row representing a different number of clusters. In the left column, data was generated by the hidden factor mechanism, where all features in a true cluster were linearly/nonlinearly linked to a latent factor. In columns 2 and 3, features in each cluster were generated using 1-dependent and 2-dependent mechanism, respectively. In such a generation mechanism, genes generated later depend on previously generated genes in the same cluster [15]. In the meantime, the first row shows results from data with 10 clusters, while the second row shows results from data with 20 clusters.

For GDHC, we used the dynamic tree cutting method [18] to cut each tree. Various values of minimum cluster size were tested. For *K*-profiles clustering, we started with a *p* value cutoff of 0.2 and gradually reduced the cutoff to 0.05 with the iterations. We ran each setting (cluster size, data generation scheme) 20 times and plotted the average results in Figure 2. We can see obviously that both *K*-profiles and GDHC outperformed linear relation-based *K*-means clustering algorithm significantly in all cluster parameter settings. *K*-profiles also did a better job than GDHC in recovering the true clusters. We allowed four minimum cluster size levels in the dynamic tree cutting, 50%, 75%, 95%, and 100%, of the true cluster size. Generally the 50% setting performed the best.

**Figure 3:**
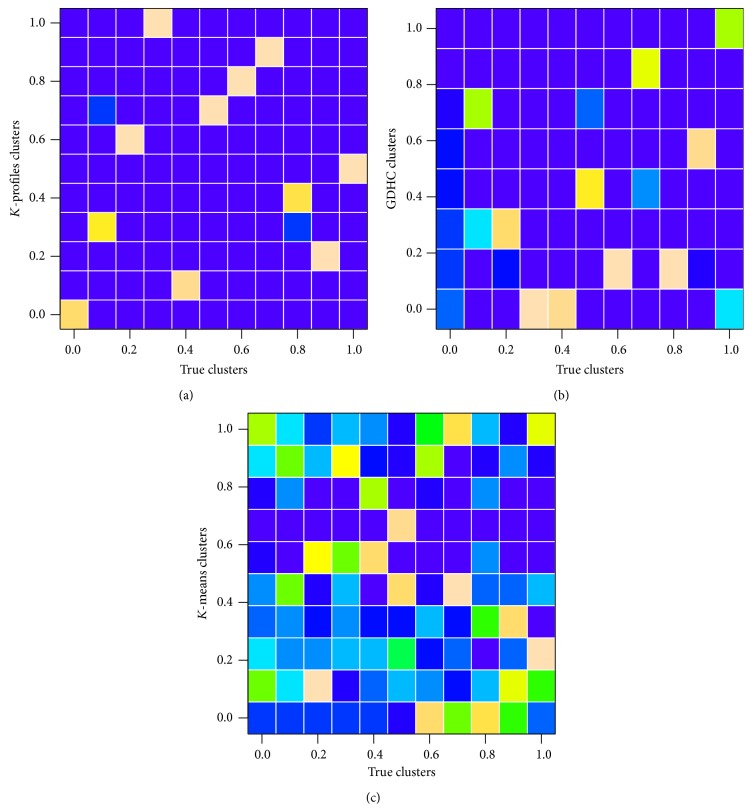
An example of confusion matrices shown as images. Cleaner pictures indicate better agreement between true clusters and clustering results. The left-most column of each subplot represents the pure noise gene group. (a) *K*-profiles clustering result. (b) GDHC result. (c) *K*-means result.

Figure 3 shows the confusion matrices of an example clustering result as images. We can see the composition of the reported clusters by the three different clustering algorithms. Cleaner images indicate better agreement between true clusters and the detected clusters. When looking into all three confusion matrices, we can see that in each reported cluster our proposed method discovered a dominant group with only a little impurity. However, in traditional *K*-means clustering, the reported clusters were mostly composed of several small groups, which rendered it of little use when the data contains much nonlinear relations. GDHC performed much better than *K*-means with 4 reported clusters (rows) composed mostly of elements from the same true clusters. Clearly, the new *K*-profiles clustering method achieved the best performance in the simulations.

**Figure 4:**
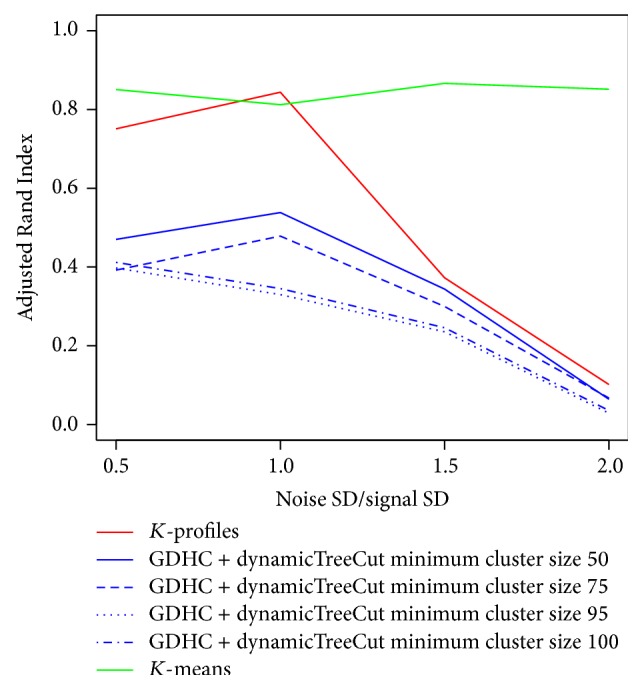
Simulation results from data with linear associations only.

The *K*-profiles and GDHC clustering methods were both based on DCOL, which detects both nonlinear and linear relationships, although it has lower power to detect linear relationship compared to correlation coefficient. Next we studied how the methods behave when the true relationships are all linear. We used the same hidden factor data generation scheme but allowed only linear relations in the data generation, which means all genes in the same cluster were linearly related to the same hidden factor. We simulated data with 10 clusters, each containing 100 genes, plus an additional 100 pure noise genes. *K*-profiles achieved similar performance to *K*-means when the noise was at low to moderate levels (Figure 4). This is likely due to the fact that *K*-means does not involve statistical testing to exclude noise genes from the clusters.

Besides being a more effective nonlinear clustering method, the *K*-profiles method is also more efficient compared to GDHC. On a data matrix with 2000 rows and 100 columns, the average computing time of *K*-profiles was ~30 seconds on a laptop with i7-3537U CPU and 6Gb memory, while the GDHC used ~600 seconds.

### 3.2. Real Data Analysis

We conducted data analysis on the Spellman yeast cell cycle data, which consists of four time series synchronized by different chemical reagents, each covering roughly two cell cycles [21]. One of the time series, the cdc15 data, contains a strong oscillating signal [22]. We removed the cdc15 dataset and used the data of the three remaining time series. The data matrix consists of 49 samples (columns) and 6178 genes (rows).

**Figure 5:**
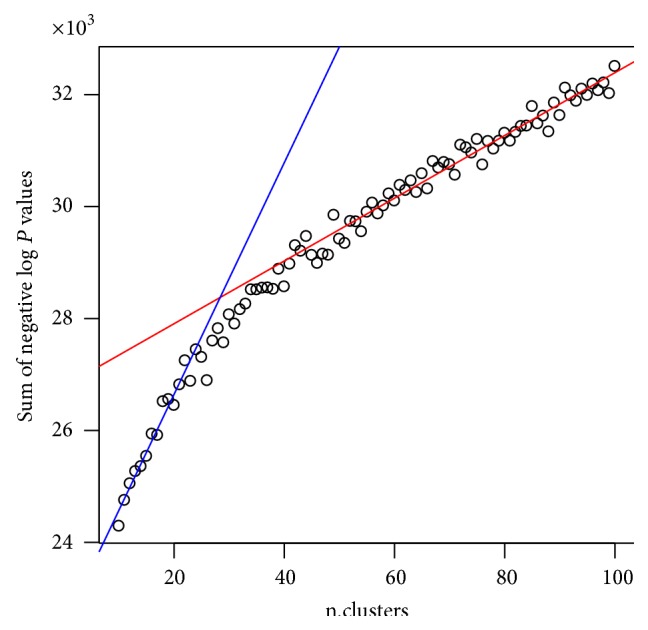
Selecting the number of clusters for the Spellman dataset by plotting sum of negative log*p* values against the number of clusters.

We applied the *K*-profiles clustering method using a series of *K* values. With each *K* value, we retained the final *p* value *p*_*i*_ of every gene. We then took the negative sum of log*p* values ∑_*i*_−log⁡(*p*_*i*_) at every *K* and plotted the value against *K*. An elbow was observed at around 30 (Figure 5). Thus we chose *K* = 30 for subsequent analyses.

**Figure 6:**
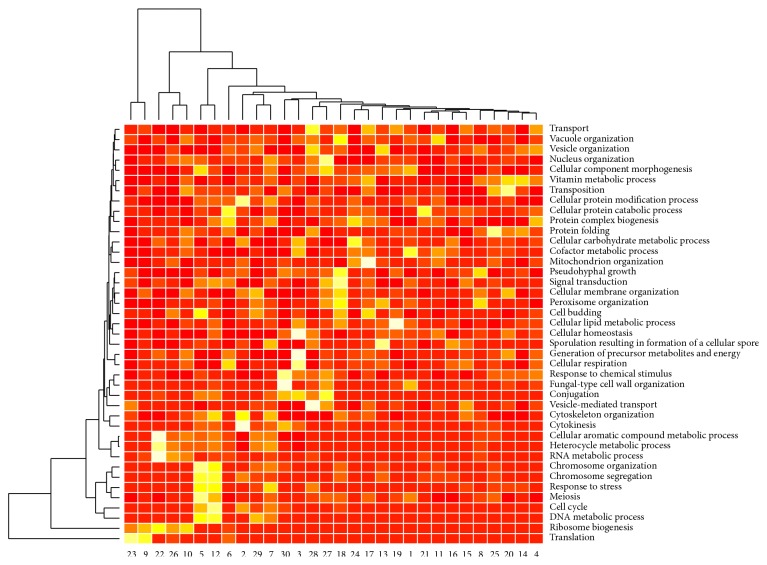
Significance levels of GO slims terms. Brighter colors indicate significance using the hypergeometric test for overrepresentation analysis.

Among the 6178 genes under study, 4874 were clustered into 30 clusters. The minimum cluster size was 59, and the maximum cluster size was 328. We then judged the performance of the methods using functional annotations. For this purpose, we resorted to Gene Ontology [23]. We used a set of GO terms that categorize genes into broad functional categories, the GO slim terms from the Saccharomyces Genome Database (SGD) [24]. Some of the GO slim terms are too broad; we limited our analysis to terms with 2000 annotated genes or less. We found that almost all the clusters are associated with certain GO slim terms using the hypergeometric test [25] for overrepresentation (Figure 6).

**Figure 7:**
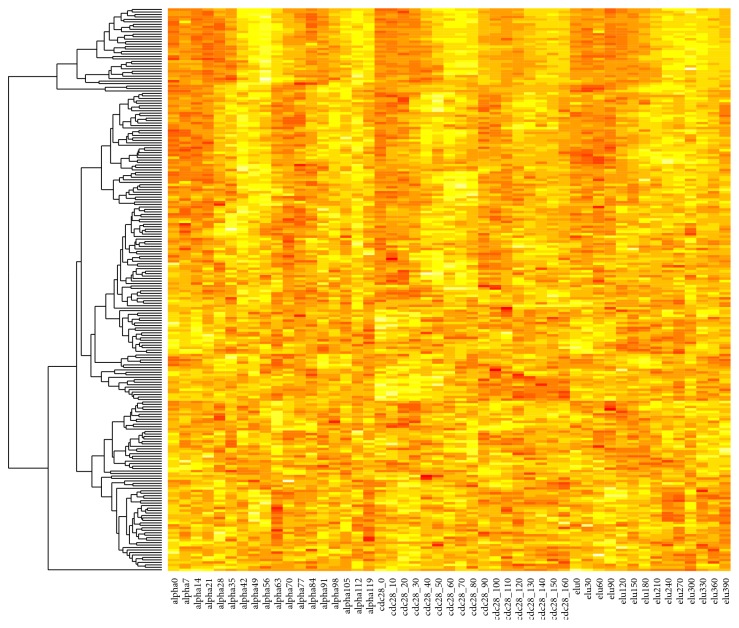
An example cluster with mostly periodically expressed genes.

From Figure 6, we see clearly that several clusters, including clusters 2, 5, 7, and 12, are highly associated with cell cycle related processes, which are clustered in the lower 1/3 region of the plot (Figure 6). We then plotted the heatmaps of the expressions of the genes in these clusters, which indeed showed strong periodical behavior. An example, cluster 2, is presented in Figure 7. We notice the genes in this cluster were mostly periodic genes, yet they exhibit different phase shifts. Such genes may not be clustered together using traditional methods based on linear associations.

The GO slim terms are broad functional categories and do not offer enough detail. We further analyzed the data using a set of 430 selected representative GO terms. The approaches to select these terms were previously described in [26, 27]. Essentially the selected terms were relatively specific, yet they were still of reasonable size. We conducted hypergeometric test for overrepresentation of these GO terms in each of the 30 clusters. We found almost all the clusters significantly overrepresent some biological processes. As examples, we show biological processes associated with clusters 2, 5, 7, and 12, which are clearly cell cycle related based on the GO slim analysis (Table 1). Many clusters clearly showed no periodical behavior. They were strongly associated with functional categories such as metabolism and signal transduction. The results are listed online at http://web1.sph.emory.edu/users/tyu8/KPC.

## 4. Conclusion

In this paper, we described a new nonlinear clustering method named *K*-profiles clustering. We incorporated statistical inference into the algorithm to remove the impact of noise genes due to their common existence in real world microarray data. The algorithm is efficient due to the quality of the Distance Based on Conditional Ordered List (DCOL). The algorithm outperformed our previous General Dependency Hierarchical Clustering (GDHC) algorithm and the traditional *K*-means clustering algorithm in our simulation studies. It generated meaningful results in real data analysis. It can be used in the analysis of high-throughput data to detect novel patterns based on nonlinear dependencies.

## Figures and Tables

**Table 1:** Biological pathways significantly associated with clusters 2, 5, 7, and 12.

Cluster	# genes	GO Biological Process ID^#^	*P* value^*^	Name of GO term
2	228	GO:0051301	1.03*E* − 07	Cell division
		GO:0006468	0.0001307	Protein phosphorylation
		GO:0010696	0.00163665	Positive regulation of spindle pole body separation
		GO:0030473	0.00584256	Nuclear migration along microtubule
		GO:0005977	0.00628021	Glycogen metabolic process

5	116	GO:0006301	5.94*E* − 06	Postreplication repair
		GO:0043570	1.87*E* − 05	Maintenance of DNA repeat elements
		GO:0006272	4.90*E* − 05	Leading strand elongation
		GO:0000070	0.00043025	Mitotic sister chromatid segregation
		GO:0009263	0.00067342	Deoxyribonucleotide biosynthetic process
		GO:0006298	0.00074914	Mismatch repair
		GO:0007131	0.00077629	Reciprocal meiotic recombination
		GO:0045132	0.00300391	Meiotic chromosome segregation
		GO:0006284	0.0034725	Base-excision repair
		GO:0006273	0.0041114	Lagging strand elongation
		GO:0006348	0.00415626	Chromatin silencing at telomere
		GO:0009200	0.00485315	Deoxyribonucleoside triphosphate metabolic process
		GO:0051301	0.00750912	Cell division

7	69	GO:0006334	4.57*E* − 12	Nucleosome assembly
		GO:0030473	6.32*E* − 05	Nuclear migration along microtubule
		GO:0030148	0.00299059	Sphingolipid biosynthetic process
		GO:0000032	0.00650292	Cell wall mannoprotein biosynthetic process
		GO:0009225	0.00774684	Nucleotide-sugar metabolic process

12	155	GO:0007020	1.07*E* − 05	Microtubule nucleation
		GO:0000070	0.0006474	Mitotic sister chromatid segregation
		GO:0006284	0.00078868	Base-excision repair
		GO:0006493	0.00078868	Protein O-linked glycosylation
		GO:0006273	0.00099378	Lagging strand elongation
		GO:0006337	0.00099378	Nucleosome disassembly
		GO:0000724	0.00151593	Double-strand break repair via homologous recombination
		GO:0000086	0.00242563	G2/M transition of mitotic cell cycle
		GO:0006368	0.00243303	Transcription elongation from RNA polymerase II promoter
		GO:0006338	0.0038366	Chromatin remodeling
		GO:0008156	0.00743106	Negative regulation of DNA replication

^#^Total number of GO Biological Process terms under study: 430.

^*^ *P* value threshold: 0.01.

## References

[1] Rung J., Brazma A. (2013). Reuse of public genome-wide gene expression data. *Nature Reviews Genetics*.

[2] Patti G. J., Yanes O., Siuzdak G. (2012). Innovation: metabolomics: the apogee of the omics trilogy. *Nature Reviews Molecular Cell Biology*.

[3] Yu T. (2010). An exploratory data analysis method to reveal modular latent structures in high-throughput data. *BMC Bioinformatics*.

[4] Zhao Y., Kang J., Yu T. (2014). A Bayesian nonparametric mixture model for selecting genes and gene subnetworks. *The Annals of Applied Statistics*.

[5] Tung A. K. H., Xu X., Ooi B. C. CURLER: finding and visualizing nonlinear correlation clusters.

[6] Jiang D., Tang C., Zhang A. (2004). Cluster analysis for gene expression data: a survey. *IEEE Transactions on Knowledge and Data Engineering*.

[7] Hastie T., Tibshirani R., Friedman J. H. (2009). *The Elements of Statistical Learning: Data Mining, Inference, and Prediction*.

[8] Gan G., Ma C., Wu J. (2007). *Data Clustering: Theory, Algorithms, and Applications*.

[9] Li K.-C., Liu C.-T., Sun W., Yuan S., Yu T. (2004). A system for enhancing genome-wide coexpression dynamics study. *Proceedings of the National Academy of Sciences of the United States of America*.

[10] Solvang H. K., Lingjærde O. C., Frigessi A., Børresen-Dale A.-L., Kristensen V. N. (2011). Linear and non-linear dependencies between copy number aberrations and mRNA expression reveal distinct molecular pathways in breast cancer. *BMC Bioinformatics*.

[11] Jian A., Zhang Z., Chang E. Adaptive non-linear clustering in data streams.

[12] Ehler M., Rajapakse V. N., Zeeberg B. R. (2011). Nonlinear gene cluster analysis with labeling for microarray gene expression data in organ development. *BMC Proceedings*.

[13] Kegl B., Krzyzak A., Linder T., Zeger K. (2000). Learning and design of principal curves. *IEEE Transactions on Pattern Analysis and Machine Intelligence*.

[14] Delicado P., Smrekar M. (2009). Measuring non-linear dependence for two random variables distributed along a curve. *Statistics & Computing*.

[15] Yu T., Peng H. (2013). Hierarchical clustering of high-throughput expression data based on general dependences. *IEEE/ACM Transactions on Computational Biology and Bioinformatics*.

[16] Yu T., Peng H., Sun W. (2011). Incorporating nonlinear relationships in microarray missing value imputation. *IEEE/ACM Transactions on Computational Biology and Bioinformatics*.

[17] Gutin G., Punnen A. P. (2002). *The Traveling Salesman Problem and Its Variations*.

[18] Langfelder P., Zhang B., Horvath S. (2008). Defining clusters from a hierarchical cluster tree: the dynamic tree cut package for R. *Bioinformatics*.

[19] Applegate D., Cook W., Rohe A. (2003). Chained Lin-Kernighan for large traveling salesman problems. *INFORMS Journal on Computing*.

[20] Hubert L., Arabie P. (1985). Comparing partitions. *Journal of Classification*.

[21] Spellman P. T., Sherlock G., Zhang M. Q. (1998). Comprehensive identification of cell cycle-regulated genes of the yeast *Saccharomyces cerevisiae* by microarray hybridization. *Molecular Biology of the Cell*.

[22] Li K.-C., Yan M., Yuan S. (2002). A simple statistical model for depicting the cdc15-synchronized yeast cell-cycle regulated gene expression data. *Statistica Sinica*.

[23] Ashburner M., Ball C. A., Blake J. A. (2000). Gene ontology: tool for the unification of biology. The Gene Ontology Consortium. *Nature Genetics*.

[24] Cherry J. M., Hong E. L., Amundsen C. (2012). Saccharomyces Genome Database: the genomics resource of budding yeast. *Nucleic Acids Research*.

[25] Falcon S., Gentleman R. (2007). Using GOstats to test gene lists for GO term association. *Bioinformatics*.

[26] Yu T., Bai Y. (2011). Improving gene expression data interpretation by finding latent factors that co-regulate gene modules with clinical factors. *BMC Genomics*.

[27] Yu T., Bai Y. (2011). Capturing changes in gene expression dynamics by gene set differential coordination analysis. *Genomics*.

